# Pore-forming peptide C14R exhibits potent antifungal activity against clinical isolates of *Candida albicans* and *Candida auris*

**DOI:** 10.3389/fcimb.2024.1389020

**Published:** 2024-03-27

**Authors:** Norida Vélez, Andreys Argel, Ann-Kathrin Kissmann, Daniel Alpízar-Pedraza, Patricia Escandón, Frank Rosenau, Ludger Ständker, Carolina Firacative

**Affiliations:** ^1^ Studies in Translational Microbiology and Emerging Diseases (MICROS) Research Group, School of Medicine and Health Sciences, Universidad del Rosario, Bogota, Colombia; ^2^ Institute of Pharmaceutical Biotechnology, Ulm University, Ulm, Germany; ^3^ Biochemistry and Molecular Biology Department, Center for Pharmaceutical Research and Development, Ciudad de La Habana, Cuba; ^4^ Microbiology Group, Instituto Nacional de Salud, Bogota, Colombia; ^5^ Core Facility for Functional Peptidomics, Faculty of Medicine, Ulm University, Ulm, Germany

**Keywords:** fungal infections, candidemia, candidiasis, antimicrobial resistance, antifungal peptides, *Candida albicans*, *Candida auris*

## Abstract

**Introduction:**

Invasive candidiasis is a global public health problem as it poses a significant threat in hospital-settings. The aim of this study was to evaluate C14R, an analog derived from peptide BP100, as a potential antimicrobial peptide against the prevalent opportunistic yeast *Candida albicans* and the emergent multidrug-resistant yeast *Candida auris*.

**Methods:**

Antifungal susceptibility testing of C14R against 99 C*. albicans* and 105 C*. auris* clinical isolates from Colombia, was determined by broth microdilution. Fluconazole was used as a control antifungal. The synergy between C14R and fluconazole was assessed in resistant isolates. Assays against fungal biofilm and growth curves were also carried out. Morphological alterations of yeast cell surface were evaluated by scanning electron microscopy. A permeability assay verified the pore-forming ability of C14R.

**Results:**

*C. albicans* and *C. auris* isolates had a geometric mean MIC against C14R of 4.42 µg/ml and 5.34 µg/ml, respectively. Notably, none of the isolates of any species exhibited growth at the highest evaluated peptide concentration (200 µg/ml). Synergistic effects were observed when combining the peptide and fluconazole. C14R affects biofilm and growth of *C. albicans* and *C. auris*. Cell membrane disruptions were observed in both species after treatment with the peptide. It was confirmed that C14R form pores in *C. albicans*’ membrane.

**Discussion:**

C14R has a potent antifungal activity against a large set of clinical isolates of both *C. albicans* and *C. auris*, showing its capacity to disrupt *Candida* membranes. This antifungal activity remains consistent across isolates regardless of their clinical source. Furthermore, the absence of correlation between MICs to C14R and resistance to fluconazole indicates the peptide’s potential effectiveness against fluconazole-resistant strains. Our results suggest the potential of C14R, a pore-forming peptide, as a treatment option for fungal infections, such as invasive candidiasis, including fluconazole and amphotericin B -resistant strains.

## 1 Introduction

The escalating global incidence of drug-resistant human pathogens represents a pressing public health concern associated with therapeutic challenges that lead to elevated morbidity and mortality rates (Fisher et al., 2022). Invasive fungal infections, particularly, are a global threat, not only because of the increase in antifungal resistance, but also because the majority of these mycoses are opportunistic, with great medical and economic impacts on individuals with compromised or weakened immune systems, such as those with HIV/AIDS, cancer, solid organ transplant recipients, among others (Kullberg and Oude Lashof, 2002; Firacative, 2020).

Invasive candidiasis, including candidemia, caused by the ascomycetous yeast species of the genus *Candida*, remains the most prevalent and life-threatening invasive fungal infection in the world (McCarty et al., 2021). In fact, despite the widespread use of antifungal prophylaxis, candidemia constitutes an important proportion of healthcare-associated bloodstream infections (BSI), being among the four most common etiologies (Kotey et al., 2021). Affecting mostly critically ill patients receiving prolonged use of broad-spectrum antibiotics and with long-term intensive care unit (ICU) stays, *Candida* infections result therefore in additional healthcare costs (Bongomin et al., 2017; Wan Ismail et al., 2020). Furthermore, the use of the current antifungal armamentarium against candidiasis is often restricted by the toxicity, drug interactions and high prices of some formulations (Perfect, 2017).

In hospital settings, *Candida albicans* is the most frequent species causing invasive infections, accounting for about half of all cases globally (Pfaller and Diekema, 2007). In Colombia, specifically, this species is reported to cause about 56% of cases (Cortes et al., 2013). Although resistance to antifungals in *C. albicans* is less common than in non-*albicans* species, the high crude and attributable mortality rates, along with the ongoing emergence of reduced susceptibility, mostly to azoles, pose significant challenges both for treatment and prophylactic strategies (Pfaller et al., 2011). *Candida auris*, on the other hand, is deemed frequently as a multidrug-resistant pathogen, difficult to identify using standard laboratory methods, which leads to delayed diagnoses and incorrect treatment (Sabino et al., 2020). Furthermore, this species is characterized by its high transmissibility and capability to persist for prolonged periods on patients’ skin and in environmental surfaces, which makes it difficult to control in healthcare facilities (Osei Sekyere, 2018). Therefore, outbreaks, as well as isolated cases of *C. auris* infections have been reported already in all continents (Jeffery-Smith et al., 2018). In Colombia, this species has caused more than 1700 cases since its first report in 2016 and it is currently the second more frequent species of *Candida* BSI in the country (Escandon et al., 2022; Ortiz-Roa et al., 2023).

The capacity of both *C. albicans* and *C. auris* to establish biofilms on medical devices, especially on central venous and urinary catheters, significantly contributes to a substantial released of yeasts into the bloodstream, hence triggering BSI (Ramage et al., 2012). These biofilms serve as a physical barrier that naturally resists the host’s immune defenses and external environmental factors (Gulati and Nobile, 2016; Sherry et al., 2017). Moreover, the formation of biofilms leads to upregulation of drug-resistance mechanisms and the development of complex regulatory processes that favor even higher levels of antifungal resistance (Ramage et al., 2012).

Together, this highlights the growing need for the development of new drugs with different mechanisms of action as well as for the search of novel approaches to treat infections caused by commonly occurring pathogenic species. During the last few decades, several antimicrobial peptides (AMPs) have been under intense investigation to be used as standalone or in combination therapies against invasive fungal infections (Rodriguez-Castano et al., 2023). These small cationic and amphipathic molecules, typically consisting of less than 50 amino acids, form part of the primary line of defense against pathogens of many organism (Matejuk et al., 2010). Among these, C14R, a designed analog derived from peptide BP100 isolated from bee venom, has previously demonstrated its antimicrobial activity against a wide range of resistant bacteria, including both Gram-positive and Gram-negative (Badosa et al., 2013; Torcato et al., 2013; Bodenberger et al., 2018; Kraemer et al., 2023). Recently, this membrane-active peptide was intensively characterized for its great antibacterial activity towards pathogenic *Pseudomonas aeruginosa* with characteristic pore forming capability hence its amphipathic structure. In addition, C14R has an overall limited toxicity against human cells with no hemolytic activity, suggesting some selectivity towards microorganisms, and has shown anti-inflammatory properties and the ability to modulate immune responses (Torcato et al., 2013; Mildenberger et al., 2024).

Therefore, this study aims to evaluate C14R as a potential new AMP against the prevalent opportunistic yeast *C. albicans* and the emergent multidrug-resistant yeast *C. auris*. Most studies evaluating the effect of AMPs in species of *Candida* have included only a few reference strains per species (Rodriguez-Castano et al., 2023). However, to our knowledge, this is the first study evaluating a large set of clinical isolates of both *C. albicans* and *C. auris*, which allows to estimate epidemiological cutoff values (ECVs) that help determining whether each species, as a population, has good or poor *in vitro* response to C14R as antifungal agent (Lockhart et al., 2017). In addition, this study shows the synergism that C14R has with fluconazole, which is broadly used against *Candida* infections (Pappas et al., 2016). The inhibitory efficacy of the peptide on biofilm formation and yeast growth, as well as its capacity on inducing a variety of cellular perturbations in both *Candida* species, is also shown. Lastly, the capability of C14R to form pores in the membrane of *C. albicans* is demonstrated.

## 2 Materials and methods

### 2.1 Isolates

Ninety-nine clinical isolates of *C. albicans* and 105 of *C. auris* were included in this study. The isolates were recovered between 2016 and 2021 from 17 departments of Colombia, as part of the National Surveillance Program of the National Institute of Health, Bogota, Colombia. Of the isolates, 157 (77%) were recovered from invasive infections, mostly BSI, while the remaining 47 (23%) were colonizing isolates, recovered from urine, urinary catheters and body parts of patients including bilateral nares, ears, axillae, groin, oral cavity, and rectum. Matrix-assisted laser desorption/ionization-time of flight mass spectrometry (MALDI-TOF MS) was used for the identification of all isolates. As reference strains per species, both clinical invasive isolates, *C. albicans* ATCC 10231 and a multidrug resistant isolate of *C. auris* H0059-13-2251 from Colombia, were included. This last isolate has a minimum inhibitory concentration (MIC) of ≥64 µg/ml for fluconazole and a MIC of 8 µg/ml for amphotericin B, as such is considered resistant to both antifungals (Escandon et al., 2022).

From the 106 C*. auris* isolates studied, 55 (51.9%) had data regarding the antifungal susceptibility against amphotericin B, given that this testing is carried out as part of the national surveillance of *C. auris* in Colombia led by the National Institute of Health (Escandon et al., 2022). From these 55 isolates, 18 (32.7%) were identified as being resistant to amphotericin B, since they had a MIC ≥2 µg/ml (CDC, 2022).

### 2.2 Peptide

C14R, with sequence CSSGSLWRLIRRFLRR and molecular weight of 2006.37 g/mol (Bodenberger et al., 2018; Mildenberger et al., 2024), was synthetized commercially by Synpeptide Co., Ltd. (Shanghai, China), and shipped in a lyophilized form with a purity of 95%. This peptide consists of seven amino acid residues that are either hydrophobic, aromatic, or non-polar, and nine that are polar or positively charged, strictly separated on opposite sides of the predicted α-helix (Mildenberger et al., 2024). At arrival, the peptide was weighed and resuspended in 4 ml of sterile water, to obtain several tubes containing a stock solution with a concentration of 400 µg/ml. These stock solutions were frozen at -20°C and thawed immediately before experiments.

### 2.3 Antifungal susceptibility testing

To determine the antifungal activity of C14R against *C. albicans* and *C. auris* clinical isolates, antifungal susceptibility testing, using broth microdilution, was carried out based on the Clinical and Laboratory Standards Institute (CLSI) guidelines, following the M27M44S protocol (CLSI, 2022). First, each isolate was cultured on Sabouraud dextrose agar (SDA) at 35°C for 24 h. From a single colony of each isolate, an inoculum, at a concentration of 1-5×10^6^ cells/ml, corresponding to the 0.5 McFarland standard, was prepared in 5 ml of sterile water. Subsequently, a 1:50 dilution was made from each cell suspension in sterile water. To achieve a final inoculum concentration of 1-5×10^3^ cells/ml, a final dilution of 1:20 was done in RPMI 1640 supplemented with 3-(N-morpholino) propanesulfonic acid (MOPS) (Sigma-Aldrich, St. Louis, MO, United States). In a 96-well round-bottom microplate, 100 µl of the final inoculum were transferred and mixed with 100 µl of each concentration of the C14R peptide, per duplicate. The range of peptide concentration, tested by 2-fold serial dilutions, was 0.390625 to 200 µg/ml diluted in RPMI-MOPs medium. The final volume in each well was 200 µl. Wells containing RPMI-MOPS alone and wells with inoculum but without the peptide were used as controls for sterility and growth, respectively. Plates were then incubated at 35°C for 24 h. The MIC of the peptide, determined visually, was defined as the lowest concentration of C14R that caused a significant decrease in growth (>50%) compared to the growth control. The antifungal susceptibility to fluconazole of all isolates was also determined following the M27M44S protocol from the CLSI (CLSI, 2022). Fluconazole (Pfizer Inc., New York, NY, USA) was used in concentrations ranging from 0.125 to 256 µg/ml. *Candida parapsilosis* ATCC 22019 and *Candida krusei* ATCC 6258 were used as reference strains, following the CLSI recommendations (CLSI, 2022). In all experiments, 20 µl of the final inoculum were plated on SDA to verify the purity of each cell suspension and colony counts, followed by incubation at 35°C for 24 h.

### 2.4 Antifungal synergism testing

To determine antifungal synergism between C14R and fluconazole, the checkerboard method, which is based on the standardized CLSI guidelines, protocol M27M44S (CLSI, 2022), was used. This method is a broth microdilution technique performed in 96-well round-bottom microplates (Spitzer et al., 2017). Considering the arrangement of the plates (8 × 12 wells) and the final volume, concentration gradients were prepared for each of the compounds utilized to create a dual concentration gradient. For the peptide, a concentration gradient ranging from 1.5625 to 200 μg/ml was tested, by dispensing, per duplicate, 50 μl of each concentration from columns 3 to 12 (from lowest to highest concentration). For fluconazole, a range between 0.25 and 128 μg/ml was tested, by dispensing 50 μl of each concentration from rows A to H (from lowest to highest concentration). To prepare the cell suspensions, each isolate was cultured on SDA at 35°C for 24 h. Thereafter, an inoculum adjusted to a concentration of 1-5×10^3^ cells/ml was prepared in RPMI-MOPS, as described above. From this suspension, 100 μl were mixed with the dual concentrations of peptide-fluconazole, to obtain a final volume of 200 μl per well. Controls for fungal growth and for sterility of the culture medium were considered in each experiment. For this assay, two clinical isolates of *C. albicans*, two of *C. auris* and the reference strain of *C. auris* H0059-13-2251, were chosen, given that, based on the antifungal susceptibility results, they had high MIC values to the peptide and were resistant to fluconazole. The reference strain of *C. albicans* ATCC 10231 was not used for this experiment, as this strain is susceptible to fluconazole and presented a low MIC to C14R.

### 2.5 Effect of C14R in biofilm formation

The capacity of C14R to inhibit the biofilm formation of *C. albicans* ATCC 10231 and *C. auris* H0059-13-2251 was assessed with two colorimetric methods. The first one, crystal violet (CV), measures biofilm biomass, while the second one, 2,3-bis-(2-methoxy-4-nitro-5-sulfophenyl)-2H-tetrazolium-5-carboxanilide (XTT) with menadione, measures metabolic activity (O'Toole et al., 2000; Jin et al., 2003). Initially, each isolate was growth on Sabouraud dextrose broth at 35°C in shaker at 100 rpm for 18 h. After centrifugation at 2500 rpm for 5 min, yeast cells were suspended in RPMI-MOPS medium and adjusted to a concentration of 1-5×10^6^ cells/ml. In a 96-well flat-bottom microtiter plate per colorimetric evaluation, CV or XTT-menadione, 100 µl of each the inoculum were transferred and mixed with 100 µl of each concentration of C14R (0.390625 to 200 µg/ml), per duplicate, and then incubated at 35°C for 24 h. After this period, the planktonic phase in the wells was aspirated, and the biofilms were washed twice with 200 μl of 1× phosphate-buffered saline (PBS). After these washing steps, the adherent cells within the biofilm were stained either with 200 µl of a 0.1% (w/v) CV solution for 15 min at room temperature or with 100 µl XTT-menadione for 2.5 h in the dark, adding the respective volume of the reagent to the wells. After staining, the solution in the plates with CV was aspirated, and the stained cells were washed twice with 200 μl of 1× PBS. Following this, the biofilms were left to dry for 24 h at room temperature. Once the plates were completely dried, the biofilms with CV were treated with 200 µl of 30% acetic acid for 15 min, after which the whole volume was transferred to a new microtiter plate. On the other hand, after staining with XTT-menadione, 80 µl of the solution were aspirated and transferred to a new microtiter plate. To finalize, the optical density (OD) of the solutions in the new plates, either with CV or XTT-menadione, was read at 560 nm and 490 nm, respectively, using an Asys Expert Plus ELISA reader (Biochrom, Ltd. Cambridge, United Kingdom). In each plate, controls include peptide-free and biofilm-free wells. Each experiment was performed three times in duplicate.

### 2.6 Growth curves

Planktonic growth rate of *C. albicans* ATCC 10231 and *C. auris* H0059-13-2251 after treatment with C14R was evaluated as reported previously (Pinilla et al., 2022). Briefly, each isolate was cultured on SDA at 35°C for 24 h, after which yeast cells were suspended in RPMI-MOPS medium to a final concentration of 1-5×10^3^ cells/ml, as described above. From the adjusted yeast suspension, 100 μl were added to plates containing 100 μl of C14R dissolved in RPMI-MOPS medium. Final peptide concentrations were adjusted to 3.125 µg/ml and 6.25 µg/ml for *C. albicans* and to 25 µg/ml and 50 µg/ml for *C. auris*, considering the antifungal susceptibility assay. These values correspond to one dilution less than the MIC values, and the MIC values of the peptide for each species, respectively. Cell suspensions of each species without the peptide were included as growth control. Subsequently, plates were incubated with orbital shaking at 100 rpm at 37°C for 48 h and OD readings at 600 nm were taken automatically every 60 minutes using the Bioscreen system (Thermo Labsystems Type FP-1100-C).

### 2.7 Evaluation of cell morphology after treatment with C14R

Cell morphology of *C. albicans* ATCC 10231 and *C. auris* H0059-13-2251 was examined following treatment with a concentration of C14R equivalent to one dilution less than the MIC values for each species, as determined by antifungal susceptibility testing. This protocol was performed as previously described (Torres et al., 2023). Briefly, each isolate was grown on SDA at 35°C for 24 h. Thereafter, a suspension of each isolate was prepared in distilled water and adjusted to a concentration of 1-5×10^6^ cells/ml. From this inoculum, 450 μl were added to a 1.5 ml Eppendorf tube, mixed with 50 μl of C14R to obtain the desired concentration (3.125 μg/ml for *C. albicans* and 25 μg/ml for *C. auris*), and incubated at 35°C for 24 h. Subsequently, cells were harvested and fixed in 2.5% glutaraldehyde (Sigma-Aldrich, St. Louis, MO, USA) for 3 h at room temperature and then in 4% paraformaldehyde (Sigma-Aldrich, St. Louis, MO, USA) for an additional hour. Cells were washed with the same solution and fixed with 1% osmium tetroxide (Sigma-Aldrich, St. Louis, MO, USA), dehydrated using an acetone gradient, and embedded in resin. Finally, the cells were stained with uranyl acetate (Thermo Fisher Scientific Inc., Waltham, MA, USA), examined in a transmission electron microscope (TEM) (JEOL JEM-1400 Plus, Tokyo, TYO, Japan), and photographed with a Gatan camera (Pleasanton, CA, USA), at the Pathology Laboratory of the Santa Fe Foundation, in Bogotá. Untreated yeasts served as controls.

### 2.8 *In silico* study of C14R-membrane interaction

#### 2.8.1 System preparation

A peptide-membrane system was generated using the Membrane Builder option CHARMM-GUI (RRID : SCR_014892) (Jo et al., 2007; Jo et al., 2008; Jo et al., 2009; Wu et al., 2014). A lipid composition mimicking the *C. albicans* membrane (Aguiar et al., 2020) was employed: 1-palmitoyl-2-oleoyl-glycero-3-phosphocholine (POPC), 1-palmitoyl-2-oleoyl-glycero-3-phosphoethanolamine (POPE), 1-palmitoyl-2-oleoyl-glycero-3-phosphoserine (POPS), 1-palmitoyl-2-oleoyl-sn-glycero-3-phosphoinositol (POPI), and ergosterol (Erg) in a ratio 59:21:3:4:13. The peptide was located at one side of the surface membrane. The TIP3P water model was used to generate explicit solvation conditions (Jorgensen et al., 1983) and the system’s charges were neutralized using a concentration of 150 mM of ions Na^+^ and Cl^-^.

#### 2.8.2 Molecular dynamics simulations

Molecular dynamic (MD) simulation was performed following a previously described protocol (Mildenberger et al., 2024), using the NAMD 2.14 package (Phillips et al., 2005) and CHARMM36 force field (Klauda et al., 2010; Vanommeslaeghe et al., 2010; Best et al., 2012). Newton’s equations of motion were integrated using the Verlet (leapfrog) algorithm (Cuendet and van Gunsteren, 2007). Periodic boundary conditions were applied in all directions. For short-range van der Waals interactions, a cutoff of 1.2 nm. At the same time, the particle mesh Ewald method (Darden et al., 1993) was applied to treat long-range electrostatic interactions, with a 1.2 nm real-space contribution cutoff for Coulombic interactions. A temperature of 310 K° and a pressure of 1 atm were maintained by the Langevin thermostat (Davidchack et al., 2009) and barostat (Zhang et al., 1995), respectively. The system was equilibrated in two steps. First, a 1000-step minimization followed by 0.5 ns of equilibration with the protein constraint was performed to guide the system to the nearest local energy minimum in configuration space. Secondly, the peptide was released from the harmonic constraints and another 0.5 ns further equilibrated the whole system. After the equilibration process, all simulations were performed for 100 ns under an isothermal-isobaric (NPT) ensemble without any restraints.

#### 2.8.3 Molecular dynamics analysis

The secondary structure contribution of the peptide in the function of time was determined using the Python library MDTraj (McGibbon et al., 2015). The distance of the peptide and its residues to the center of the membrane, and the surface interaction area were calculated using *in house* developed codes implemented in the software Visual Molecular Dynamics (Humphrey et al., 1996). The interaction analysis was performed in the last 10 ns of the simulation using the hydrogen bonds plugin of VMD and the Python library ProLIF (Bouysset and Fiorucci, 2021).

### 2.9 C14R permeabilization assay

To experimentally demonstrate pore-formation capability of C14R in the *C. albicans* membrane, a permeabilization assay was performed as described before (Mildenberger et al., 2024). For this, four fluorescent dyes were used, namely fluorescein (FITC), propidium iodide, ATTO 488 alkyne and rhodamine phalloidin (Thermo Fisher Scientific Inc., Schwerte, Germany) at final concentration of 5 µl/ml in 1× PBS. These dyes have different molecular sizes ranging from 389 to 1250 Da (FITC < propidium iodide < ATTO 488 alkyne < rhodamine phalloidin). In brief, 10^7^ C*. albicans* ATCC 90028 cells in 200 µl RPMI broth supplemented with 12 µg/ml C14R were incubated at 37°C for 2 h. Ten minutes prior to the end of the 2 h incubation time, 100 µl of a 0.1% (w/v) solution of Triton X-100 (Sigma-Aldrich, St. Louis, MO, USA) was added and served as positive permeation control. After incubation, the cells were centrifuged at 11000 × g and the supernatant was discarded, washed with 1× PBS, and after the addition of 5 µl each fluorescent dye and 195 µl 1× PBS for 20 min, the cell suspension was centrifuged at 11000 × g for 2 min, respectively. Supernatants were discarded, and then the cells were fixated for 10 min using 4% (w/v) solution of paraformaldehyde (Carl Roth GmbH, Karlsruhe, Germany). Subsequently, the yeasts cells were washed three times with 1× PBS, resuspended in 200 µl 1× PBS, and transferred to a flat-bottomed polystyrene 96 wells microtiter plate. Fluorescence measurements were conducted at excitation wavelengths of 498 nm (FITC), 535 nm (propidium iodide), 500 nm (ATTO 488 alkyne), 540 nm (rhodamine phalloidin), and emissions of 517 nm (FITC), 617 nm (propidium iodide), 520 nm (ATTO 488 alkyne), and 565 nm (rhodamine phalloidin) with a Tecan SPARK microplate reader (Tecan Group Ltd., Männedorf, Switzerland). Additionally, microscopic analyses were performed at 630× magnification using a Leica DMi8 fluorescence microscope (Leica Microsystems CMS GmbH, Wetzlar, Germany). Cells remained untreated for negative controls.

### 2.10 Statistical analysis

For each species, the frequency of MICs, mode, and geometric mean MICs of C14R and of fluconazole were determined. Differences in MICs between invasive and colonizing isolates, per species, were established using a Mann-Whitney test. The Pearson correlation coefficient (ρ) was used to assess the association between the MIC values of the peptide and those of fluconazole. ECVs for C14R, per species, were estimated with ECOFFinder (RRID : SCR_018149), a freely available Microsoft Excel spreadsheet calculator, following the methodology described previously (Turnidge et al., 2006). ECOFFinder was also used to generate graphs of the MIC distributions per species. This method has been adopted by the CLSI as a standard method for ECV determination (CLSI, 2016; CLSI, 2022). Definition of fluconazole and amphotericin B resistance in *C. auris* was done according to the interpretation of the Centers for Disease Control and Prevention (CDC), while fluconazole resistance in *C. albicans* was defined following the clinical breakpoints established by the CLSI (CDC, 2022; CLSI, 2022).

In checkerboard tests, to assess synergism, numerous combinations of antimicrobial agents were evaluated, and conventionally, the fractional inhibitory concentration (FIC) index value from the most effective combination was calculated using the formula: FIC = (MIC of drug A in combination/MIC of drug A alone) + (MIC of drug B in combination/MIC of drug B alone). The combination of antifungal agents was considered synergistic when FIC ≤0.5 μg/ml, and antagonistic when FIC >4.0 μg/ml, and any value in between was interpreted as indifferent (Maurya et al., 2011).

Statistical analyses, growth curves and molecular dynamics simulations graphs were performed with GraphPad Prism 9 (RRID: SCR_002798) and Microsoft^®^ Excel^®^ (RRID : SCR_017294). When established, *p*-values <0.05 were considered statistically significant.

## 3 Results

### 3.1 C14R has potent *in vitro* antifungal effect against clinical isolates of *C. albicans* and *C. auris*

**Figure 1:**
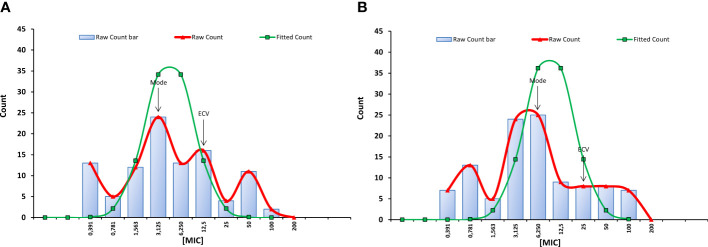
Minimal inhibitory concentrations (MIC) distributions of C14R against 99 clinical isolates of *Candida albicans* from Colombia and the reference strain of *C. albicans* ATCC 10231 **(A)** and against 106 clinical isolates of *Candida auris* from Colombia **(B)**. Modes and ECVs 95% are indicated. Values in X axis are in µg/ml.

The geometric mean MIC of C14R against the 99 clinical isolates of *C. albicans* plus the reference strain of the species was 4.42 µg/ml, with a mode that was the fourth lowest concentration tested (3.125 µg/ml) with 24 isolates. In addition, the ECV 95% for the species was 12.5 µg/ml, which is only two dilutions more than the mode (**Figure 1A**). Altogether, this indicates that C14R has very good antifungal activity for clinical isolates of *C. albicans*.

Regarding *C. auris*, it was possible to determine that C14R has also a good antifungal activity for the species, although this was to some extent inferior to that observed against *C. albicans* isolates. Both the mode (6.25 µg/ml) and the ECV 95% (25 µg/ml) of C14R against the 105 clinical isolates of *C. auris* plus the reference strain of the species (**Figure 1B**) were one dilution higher than the mode and the ECV 95% of *C. albicans*. In addition, the geometric mean MIC (5.34 µg/ml) was slightly higher. However, statistically, the susceptibility of the isolates of each species to the peptide did not differ among species (*p* = 0.3752).

Notably, none of the isolates, neither of *C. albicans* nor of *C. auris*, was able to grow in the highest concentration of C14R tested (200 µg/ml). In addition, statistically, the susceptibility of the isolates of each species to the peptide did not differ according to the source of the isolates. The geometric mean MICs of invasive isolates compared to colonizing isolates was 4.77 µg/ml *vs.* 3.74 µg/ml for *C. albicans* (*p* = 0.4267) and 5.78 µg/ml *vs.* 3.53 µg/ml for *C. auris* (*p* = 0.2193), respectively.

While C14R displayed a potent antifungal activity against clinical isolates of both *C. albicans* and *C. auris*, the antifungal activity of fluconazole was much lower against *C. auris*. Not only the geometric mean MICs of this azole differ statistically among *C. albicans* and *C. auris* (0.58 µg/ml *vs.* 9.94 µg/ml, *p <*0.0001), but also the mode (0.125 µg/ml *vs.* 2 µg/ml, *p <*0.0001). In addition, 36 (34%) isolates of *C. auris* were identified as fluconazole resistant (MIC ≥32 µg/ml), while most isolates (85%) of *C. albicans* were identified as susceptible (MIC ≤2 µg/ml). In *C. albicans*, three isolates were susceptible dose-dependent (SSD) (MIC = 4 µg/ml) and 12 were resistant (MIC = 64 µg/ml) to fluconazole. No association was found between the susceptibility of *C. albicans* to C14R and fluconazole (ρ = 0.04598, *p* = 0.6497), nor in the susceptibility of *C. auris* to both agents (ρ = -0.1617, *p* = 0.0978). However, from the 36 fluconazole-resistant *C. auris* isolates, four (11.1%) had simultaneously a high MIC to C14R (≥50 µg/ml).

From the 18 C*. auris* isolates that were resistant to amphotericin B, nine (50%) had in addition concomitant resistance to fluconazole. However, from this multidrug-resistant isolates, eight had a low MIC (≤6.25 µg/ml) to C14R and only one presented a MIC of 50 µg/ml to the peptide, which indicates that the peptide has very good antifungal activity against these fluconazole-amphotericin B resistant *C. auris* isolates.

The MIC values for C14R and fluconazole against the reference strains of *C. albicans* ATCC 10231 were 6.25 µg/ml and 1 µg/ml, respectively, and for *C. auris* H0059-13-2251 were 50 µg/ml and 128 µg/ml, respectively.

### 3.2 C14R has synergistic effect with fluconazole against clinical isolates of *C. albicans* and *C. auris* resistant to this azole

Among the studied clinical isolates, two of *C. albicans* and three of *C. auris* presented high MIC values to C14R (≥50 µg/ml) and were concomitantly resistant to fluconazole (≥32 µg/ml) (**Table 1**). However, when the isolates were grown with different combinations of these agents, a clear synergistic effect was noticed, as the MIC values of C14R decreased between two and five dilutions and the MIC values for fluconazole decreased between one and six dilutions, with most FIC indexes ≤0.5 μg/ml (**Table 1**). Notably, the combinations of C14R and fluconazole tested with each isolate caused the complete killing of *Candida* cells in both species.

**Table 1:** Synergistic effect of C14R and fluconazole against clinical isolates of *Candida albicans* and *Candida auris* from Colombia resistant to fluconazole.

Species	Isolate	Minimal inhibitory concentration (μg/ml)					FIC index (μg/ml)
		C14R		FCZ			
		Alone	Combined	Alone		Combined	
*C. albicans*	H0059-1-137	50	1.5625	64		2	0.34
	H0059-1-146	50	1.5625	64		2	0.34
*C. auris*	H0059-13-009	100	3.125	32		16	0.53
	H0059-13-464	50	1.5625	64		8	0.44
	H0059-13-2251	50	12.5	128		2	0.27

FIC, fractional inhibitory concentration; FCZ, fluconazole.

Minimal inhibitory concentration (MIC) values of C14R alone or in combination with fluconazole are shown, as well as the MIC values of fluconazole alone or in combination with C14R.

### 3.3 C14R is effective in eradicating *C. albicans* and *C. auris* biofilms

**Figure 2:**
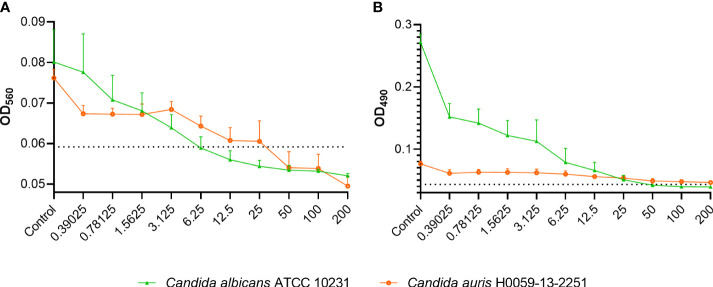
Effect of C14R on the formation of biofilm of *Candida albicans* ATCC 10231 and *Candida auris* H0059-13-2251. The biomass **(A)** and the metabolic activity **(B)** are shown. Yeasts growing without C14R were used as control. The absorbance of the culture media without yeasts and the peptide is indicated by a dotted line. Values in X axis are in µg/ml.

Treating *C. albicans* ATCC 10231 biofilms with 6.25 µg/ml of C14R, which equals the MIC, not only decreased the total biofilm biomass (**Figure 2A**) but it was also effective in killing about 85% of the yeasts embedded in the biofilms (**Figure 2B**). Similarly, in *C. auris* H0059-13-2251, a concentration of about 25 µg/ml of C14R, which is one dilution less than the MIC, decreased almost the total biofilm biomass (**Figure 2A**) and it was effective in killing about 90% of the yeasts embedded in the biofilms (**Figure 2B**). Notably, *C. albicans* is able to produce more biofilm that *C. auris*, as it has been reported before (Sherry et al., 2017).

### 3.4 C14R inhibits the growth of *C. albicans* and decreases the growth of *C. auris*

**Figure 3:**
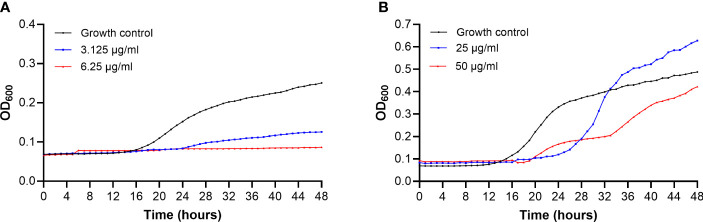
Growth curves of *Candida albicans* ATCC 10231 **(A)** and *Candida auris* H0059-13-2251 **(B)** after treatment with C14R. Yeast growing without treatment were used as growth control (black line). Growth of yeasts treated with one dilution less than the MIC values (blue line) and the MIC (red line) of the peptide for each species, are shown.

*C. albicans* growth is completely inhibited when is treated with a concentration of C14R that equals the MIC (6.25 µg/ml), and it is much slower when is treated with one dilution less than the MIC (3.125 µg/ml), which suggests that the peptide might be fungicide against this species (**Figure 3A**). Without treatment, the cell suspension of *C. albicans* reaches double the OD in ~23 hours with a constant increase in turbidity, while with the lower dose of treatment (3.125 µg/ml), the growth of *C. albicans* does not double even after 48 hours.

Compared to the effect on *C. albicans*, C14R shows a less strong effect on *C. auris*, given that the growth of the last species is not completely inhibited by the peptide. During the first 24 hours, *C. auris* treated with 25 µg/ml or 50 µg/ml grows much slower than without treatment. However, while the growth of *C. auris* at a concentration of C14R that equals the MIC (50 µg/ml) remains lower to untreated cells, even after 48 hours, a smaller concentration of the peptide (25 µg/ml) does not affect the yeasts growth after 27 hours, but instead the growth of treated cells become higher than the growth control, which suggests that the peptide might be fungistatic against *C. auris* (**Figure 3B**).

### 3.5 C14R causes cell perturbations to *C. albicans* and *C. auris*

**Figure 4:**
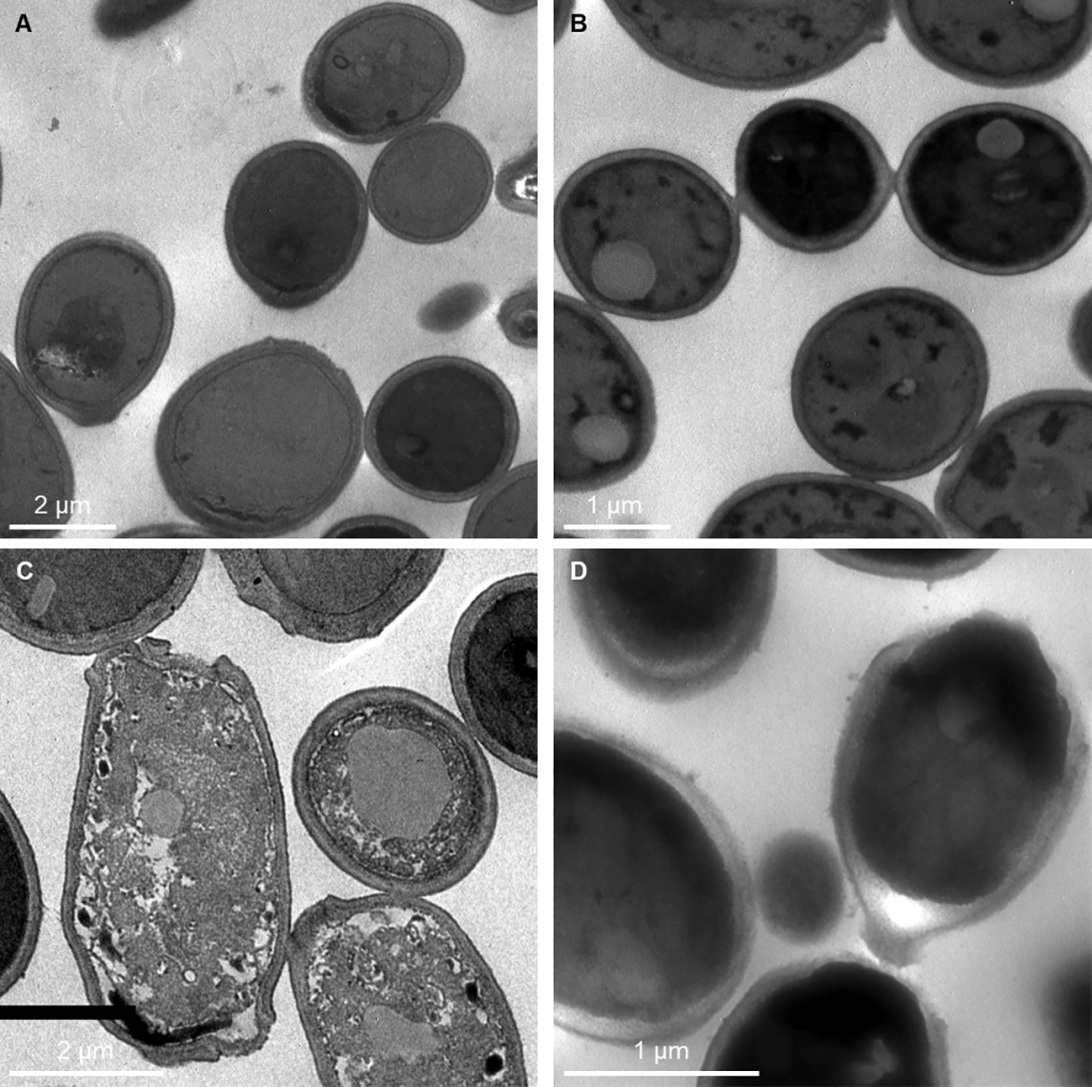
Transmission electron photographs showing *Candida albicans* ATCC 10231 and *Candida auris* H0059-13-2251 cell damage by C14R. Untreated cells of *C. albicans* **(A)** and *C. auris* **(B)** have intact cell wall and cell membranes with uniform cytoplasm. *C. albicans* treated with 3.125 μg/ml of C14R **(C)** and *C. auris* treated with 25 μg/ml of C14R **(D)** lost their regular spherical shape by disruption of the membrane with altered cytoplasm.

After treatment with C14R, the ultrastructure of both *C. albicans* ATCC 10231 and *C. auris* H0059-13-2251 was clearly changed. In untreated cells, the cell wall was smooth, the structures of the cell wall and cell membrane were intact, and the cytoplasm was uniform and full (**Figures 4A, B**). In treated cells, disruption of the membrane, causing the cell to lose its regular spherical shape, was observed in both species (**Figures 4C, D**). In addition, particularly in *C. albicans*, the surface of the cell seemed rough and there were white spots in the cells, which make the cytoplasm looks uneven with a disordered cytoplasmic structure (**Figure 4C**).

### 3.6 C14R forms pores in *C. albicans*

**Figure 5:**
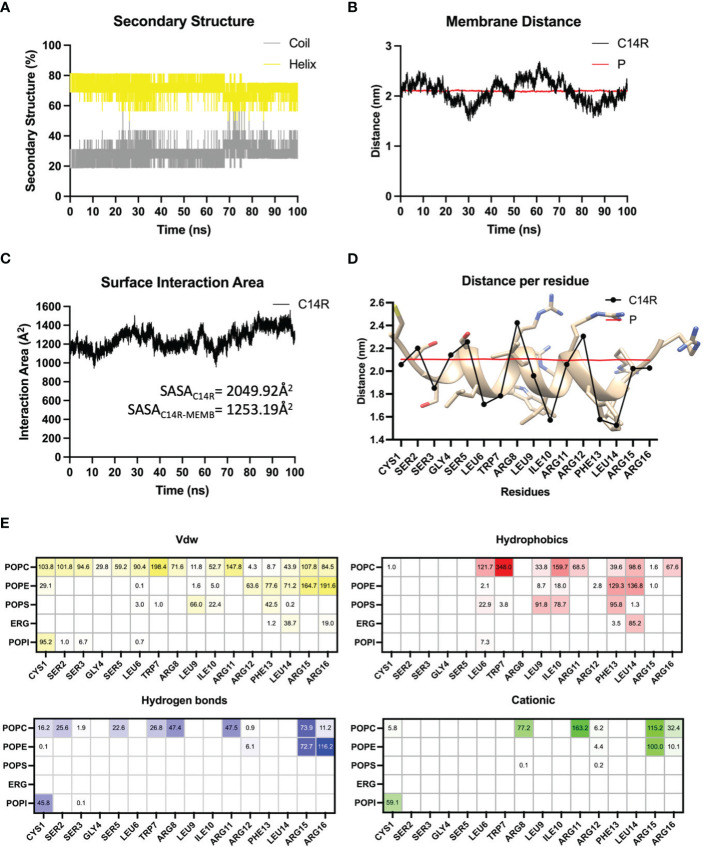
Molecular dynamics simulations analysis evaluating the secondary structure contribution **(A)**, the distance of the peptide to the center of the membrane **(B)**, and the surface interaction area of C14R **(C)**, in function of time. The average distance of each residue to the center of the membrane evaluated **(D)** as well as the interaction decomposition between each residue of the peptide with membrane’s lipids **(E)** in the last 10 ns of simulation, are shown. Values represent the occupancy of the interactions in percentage, those residues with more than 100% indicate that it was able to interact with more than one lipid at the same time.

A lipid composition mimicking the *C. albicans* membrane was simulated and served to assess anti-*Candida* C14R interactions. C14R has a net charge of 5+ provided by five positively charged residues, constituting the 31.25% of the sequences. This feature drives the first step into the mechanism of action of AMPs adsorption. Here, the electrostatic forces between the positively charged residues of C14R and the negative charge of the lipid’s phosphate heads play an essential key role. Once the peptide has adsorbed onto the membrane, it was located at one side of the surface membrane and it presented elevated structural stability during the whole molecular dynamics’ simulation with a high contribution of α-helix (~75%) and a lower contribution of disordered structure (~25%) (**Figure 5A**). Additionally, it showed a tight association with the membrane, being located at the water-membrane interface, and at some points of the simulation (20 ns to 40 ns and 75 ns to 100 ns) crossing the phosphates barrier (**Figure 5B**). During the 100 ns of simulation, the average surface area of the peptide interacting with the membrane was 1253.19Å^2^ representing 61.13% of the total area (2049.92 Å^2^) of the peptide (**Figure 5C**), where it adopted a parallel orientation to the membrane surface locating its polar residues Arg and Ser to the solvent and more hydrophobic residues as Leu, Phe, and Ile, deeply buried in the membrane (**Figure 5D**). The orientation adopted by the peptide on the membrane of *C. albicans* ATCC 90028 facilitates the interaction of all residues by short-range Van der Walls interactions. Also, the amphipathic nature of C14R and the insertion of hydrophobic residues into the membrane allow the peptide-membrane complex stabilization by hydrophobic interactions mostly between Leu6, Trp7, Leu9, Ile10, Phe13, and Leu14, and the lipid’s tails. On the other hand, polar residues exposed to the solvent and in the interface can establish several hydrogen bond interactions, especially Cys1, Ser2, Ser5, Trp7, Arg8, Arg11, Arg15, and Arg16. In addition, most of the Arg residues and Cys1 are involved in cationic interaction through their positive charge and the negative charge of the lipid’s phosphate heads (**Figure 5E**). The fact that arginines are highly involved in both interactions, hydrogen bonds and cationic interactions, reinforces the idea that these positive charges are highly involved in driving the peptide toward the negative surface of *C. albicans* and its further stabilization through electrostatic forces.

**Figure 6:**
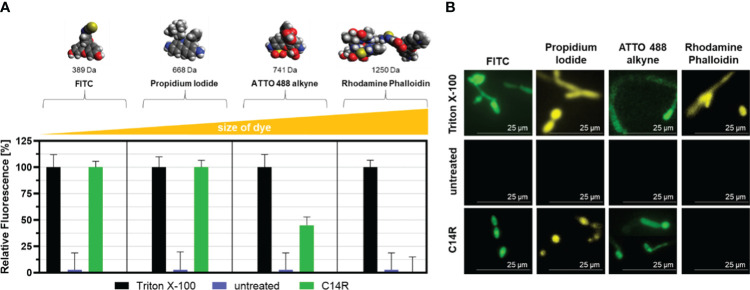
Permeabilization assay of *Candida albicans* ATCC 90028 membrane after treatment with C14R. Staining of porous cells using fluorescein (FITC), propidium iodide, ATTO 488 alkyne, or rhodamine phalloidin as fluorescent dyes with Triton X-100 serving as positive control agent **(A)**. Experiments were conducted in triplicate and error bars represent standard deviations. Fluorescence microscopy analysis of *C. albicans* cells treated with Triton X-100 and C14R showing internalized dyes is also shown at 630× magnification **(B)**.

In addition, the predicted pore-forming ability of C14R, assessed by a permeabilization assay, showed that the detergent Triton X-100 served as the ultimately pore-forming positive control agent (**Figure 6A**). While FITC and propidium iodide were able to enter the cells unhindered after treatment with C14R, ATTO 488 alkyne could only enter to a certain degree and rhodamine phalloidin was completely excluded from entering the cells (**Figure 6A**). This shows that the peptide not only forms pores, but also defines a certain size limit for molecules to enter the cytoplasm via this introduced gates. By fluorescence microscopy it was possible to verify that Triton X-100 permeabilized *C. albicans* cells perfectly, allowing all dyes to be internalized, while C14R-treated cells allowed the entrance only of FITC, propidium iodide and, to a lesser extent, ATTO 488 alkyne (**Figure 6B**).

## 4 Discussion

*C. albicans*, one of the most common fungal pathogens affecting critically ill individuals, and *C. auris*, for its multidrug resistance and ability to persist in the patient’s body and hospital’s environments, are species with treatment and management challenges. As such, these yeasts were currently ranked as critical in the fungal priority pathogens list of the World Health Organization, to guide research, development, and public health action (WHO, 2022). Therefore, the evaluation of new molecules, such as the peptide C14R, as potential alternative therapies against these opportunistic pathogens, is relevant and opportune, given that newer agents with novel mechanisms of action can possibly overcome the limitations of the currently available arsenal of antifungals (Mba and Nweze, 2022; Firacative, 2023).

It is known that AMPs are crucial players in innate immunity, inhibiting microorganisms through membrane disruption and targeting intracellular structures (Hancock and Sahl, 2006; Matejuk et al., 2010). Moreover, acquired resistance to these molecules is rare regardless of prolonged use (Bechinger and Gorr, 2017). Thus, the antimicrobial activity of diverse peptides has been proved against different human pathogens, such as C14R and peptide derivatives against resistant bacteria, including Gram-negative species like *Escherichia coli*, *Klebsiella pneumoniae*, *Pseudomonas aeruginosa*, and Gram-positive species like *Staphylococcus aureus*, *Streptococcus pneumoniae*, and *Enterococcus faecium* (Torcato et al., 2013). However, this is the first study evaluating the activity of C14R against fungi, specifically *C. albicans* and *C. auris*. Importantly, the peptide’s antifungal activity was not only assessed against one reference strain per species, but against a large number of clinical isolates per species, including invasive and colonizing ones, as well as fluconazole and amphotericin B resistant isolates. This study expands our understanding of the antimicrobial action of C14R against diverse microorganisms of public health concern.

The determination of ECVs per species allowed us to infer that C14R has a very good activity against *C. albicans* and *C. auris*, as populations, considering that the concentration of the peptide that is able to inhibit the growth of >95% of the isolates, was significantly lower than the MIC values reported in other studies evaluating AMPs against these pathogens (Vicente et al., 2019; Cheng et al., 2021; Gonzalez-Garcia et al., 2021; Jayasinghe et al., 2023; Rodriguez-Castano et al., 2023). The MIC mode of each species, which was the fourth (3.125 µg/ml) and fifth (6.25 µg/ml) lowest concentrations tested, showed us that an important proportion of isolates can be considered susceptible to C14R, suggesting its potential as a therapeutic option against infections caused by either *Candida* species. Notably, no association was found between the susceptibility to C14R and the susceptibility to fluconazole, which suggests the potential efficacy of the peptide against isolates that are resistant to this azole, as well as against multidrug-resistant isolates, like it is the case of *C. auris* isolates exhibiting in addition resistance to amphotericin B.

Even in the small subset of fluconazole-resistant *C. albicans* and *C. auris* isolates that concomitantly exhibited higher MICs to C14R, a synergistic effect between both molecules was observed. Considering that the use of antifungals as monotherapy is often limited by their toxicity, drug interactions, the need for intravenous administration, and in many cases, affordability, alternative approaches are required, particularly those combining two antifungals, which enhances efficacy of each drug alone and decrease resistance (Vitale, 2021; Mba and Nweze, 2022). Therefore, C14R could be eventually used not only alone but also in combination with fluconazole, which is one of the antifungals commonly used in the treatment of *Candida* infections (Pappas et al., 2016), in order to improve its therapeutic activity and reduce its doses. Further studies, including an *in vivo* model of invasive candidiasis, are needed to optimize dosing regimens to use C14R as an adjunctive therapy.

Apart from the antifungal activity of C14R assessed by broth microdilution, it was possible to establish this peptide as another effective molecule for eradicating the biofilms formed by both *C. albicans* and *C. auris* (Kubiczek et al., 2020; Amann et al., 2022; Haring et al., 2022; Amann et al., 2023). Even though the formation of biofilms is a dynamic and complex process, it is known that biofilms contribute significantly to persistent foci, recurrent candidemia and antifungal resistance, highlighting the further potential of C14R to improve therapeutic efficacy (Ramage et al., 2012; Pierce et al., 2017). Particularly in *C. albicans*, which has shown to be a much stronger biofilm producer that *C. auris* (Sherry et al., 2017), the diminution in biofilm biomass and the reduction of cell viability of the yeasts were significant after treatment with C14R. Considering that biofilm formation is a key driver of *C. albicans* pathogenicity, we emphasize the utility of AMPs to treat high biofilm-forming species, whose infections have been associated with poorer clinical outcomes and higher mortality rates (Tumbarello et al., 2012; Gulati and Nobile, 2016).

C14R showed as well to cause significant morphological and structural alterations in both *C. albicans* and *C. auris* cells, thus, their growth was also affected. The mechanism of action of C14R involves its interaction with the cell membrane of the target microorganisms (Torcato et al., 2013). Particularly, the formation of pores seems to be a surprisingly simple mode-of-action for AMPs, as the activity mainly depends on the diversion of the hydrophobic and hydrophilic amino acids residues on the opposite sides of the molecules (Huang et al., 2010). The recently developed amphipathic peptide C14R perfectly follows nature’s design concept for an α-helical molecule with the concentration of hydrophobic residues facing one side of the helix and polar residues residing on the opposite side (Mildenberger et al., 2024). This specific structural pattern is crucial for the peptide’s activity, as the cationic polar domain is essential for the initial interaction with the surface of the bio-membrane, whereas the hydrophobic patch drives the insertion into the membrane core of the hydrocarbon chain, primarily mediated by van der Waals and hydrophobic interactions (Teixeira et al., 2012). Here we revealed that C14R has in fact the ability to penetrate a model membrane composed according to the lipid “recipe” of a typical *C. albicans* cell. Moreover, we proved the capability of C14R to form peptide aggregates as amphipathic pore-like structures in the phospholipid bilayer of the target bio-membrane. Although only the two smallest molecules (389 and 668 Da) were taken up perfectly and the third (741 Da) being partially internalized upon C14R exposition, it can be suggested that the peptide-based pore possesses a molecular size cut-off in this range, but this is currently not completely understood and further investigations are needed to prove whether it is really only a size cut-off or if the chemical properties of the fluorophores may influence their uptake. However, from the experimental results, it can be concluded that the hydrophilic regions of the peptide line the interior of the pore and form a water-filled channel, which thereby disrupts the barrier function of the lipid bilayer of the membrane and hence allows the passage of the fluorescent dyes. Upon pore-formation the integrity of the cell membrane is disrupted, resulting in leakage of cell contents, loss of membrane potential, and consequently cell death (Huan et al., 2020).

Our results show that C14R has a potent antifungal activity against clinical isolates of both *C. albicans* and *C. auris*. This antifungal activity remains consistent across isolates regardless of their clinical source, invasive or colonizing, and their antifungal susceptibility profile to fluconazole and amphotericin B. Notably, the combination of the peptide with fluconazole resulted in a synergistic effect against resistant isolates. We also showed the capacity of C14R to affect formed biofilms, and growth of *C. albicans* and *C. auris*, with clear cell membrane disruptions in both species, most likely due to its capacity to form pores in the membrane. Together, the results of this study demonstrate the potential of C14R as a treatment option against *C. albicans* and *C. auris*, including multidrug resistant strains. The evaluation of diverse AMPs, such as C14R, as possible alternatives to effectively manage and address the global issue of antifungal resistance is a key step forward to advance in the ability to treat patients with invasive mycoses such as candidiasis. Further research can be undertaken to evaluate the activity of C14R in an *in vivo* model of infection, considering that one of the main challenges of the clinical use of AMPs is their low bioavailability, given that these molecules can be degraded by mammalian proteases. Nevertheless, the negative feature of peptide degradation can be overcome by different strategies that improve AMP pharmacokinetics, including chemical modifications and delivery systems such as nanoparticles (Botelho Sampaio de Oliveira et al., 2023).

## Data availability statement

The raw data supporting the conclusions of this article will be made available by the authors, without undue reservation.

## Author contributions

NV: Data curation, Formal analysis, Investigation, Methodology, Writing – original draft. AA: Investigation, Writing – review & editing. A-KK: Data curation, Investigation, Methodology, Writing – review & editing. DA-P: Data curation, Investigation, Methodology, Writing – review & editing. PE: Conceptualization, Resources, Writing – review & editing. FR: Conceptualization, Resources, Supervision, Writing – review & editing. LS: Conceptualization, Funding acquisition, Project administration, Resources, Writing – review & editing. CF: Conceptualization, Formal analysis, Funding acquisition, Methodology, Project administration, Resources, Supervision, Writing – review & editing.
